# Correction to ‘Interest in non-social novel stimuli as a function of age in rhesus monkeys’

**DOI:** 10.1098/rsos.200316

**Published:** 2020-04-22

**Authors:** Eliza Bliss-Moreau, Mark G. Baxter

*R. Soc. open sci.* **6**, 182237. (Published Online 27 September 2019). (doi:10.1098/rsos.182237)

Shortly after the publication of our article, it was brought to our attention that there was an error in the R code for our analyses that resulted in ‘Day’ being treated as a numerical variable with values of 1 or 2 instead of as a categorical variable in linear mixed models analyses. The graphical displays of data in our article are correct, but the statistical results presented in the text of the Results section reflect numerical rather than categorical coding of the Day variable. Corrected R code and statistical results have been added to the DataDryad archive associated with this paper (https://doi.org/10.5061/dryad.1bj133v). Correcting this error did not affect the results of analyses of exploration of the novel object during the first 2 min, which replicated the procedure of Almeling *et al.* (ref. 11 in the original article). However, for analyses of the entire 20 min test period, there are now significant main effects of age from the linear mixed models analyses: for counts of how many of the bins had any activity, *F*_1, ∼240.18_ = 5.27, *p* < 0.0005; for total amount of food lost during the test period, *F*_1, 241_ = 10.91, *p* = 0.001; for total activity counts across the entire 20 min, *F*_1, ∼241.23_ = 11.78, *p* = 0.0007. The latter analysis also still had a significant interaction of age and test day, *F*_1, ∼240.58_ = 8.26, *p* = 0.004, as we reported in the original paper.

These updated analyses do not change our main conclusions that we failed to obtain the same results as Almeling *et al.* in a conceptual replication of their work, although it appears that with prolonged exploration of a novel object (in this case a food puzzle shaker) over multiple test days and time within a session, older monkeys tend to lose interest sooner than younger monkeys do.

